# A case report: delayed high fever and maculopapules during Sorafenib treatment of ectopic hepatocellular carcinoma

**DOI:** 10.1186/s12885-016-2590-9

**Published:** 2016-07-27

**Authors:** Tianxiang Cui, Xinwei Diao, Xiewan Chen, Shaojiang Huang, Jianguo Sun

**Affiliations:** 1 Cancer Institute of PLA, Xinqiao Hospital, Third Military Medical University, Chongqing, 400037 China; 2 Department of pathology, Xinqiao Hospital, Third Military Medical University, Chongqing, 400037 China; 3 Medical English Department, College of Basic Medicine, Third Military Medical University, Chongqing, 400038 China

**Keywords:** Sorafenib, Delayed reaction, High fever, Maculopapules, Ectopic hepatocellular carcinoma

## Abstract

**Background:**

Sorafenib is the standard first-line therapy for hepatocellular carcinoma (HCC) and probably ectopic hepatocellular carcinoma (EHCC) as well. No report involves a side effect of delayed high fever of sorafenib. This manuscript describes a case of EHCC in the thoracic and abdominal cavities, who showed a delayed high fever and maculopapules during sorafenib treatment.

**Case presentation:**

The patient is a 63-year-old Chinese male with advanced EHCC, taking sorafenib 400 mg twice daily. On the tenth day, red maculopapules appeared all over the body. On the same day, the patient began to suffer from continuous high fever. Due to these effects, the patient was asked to cease sorafenib treatment, and the high fever and maculopapules were alleviated quickly. However, the symptoms were present again upon re-challenge of sorafenib. Prednisone was then administered to control the symptoms, with the dosage gradually reduced from 30 to 5 mg/day in 1.5 months. No recurrence of fever or maculopapules has been found. Tumor response reached partial response (PR) and progression free survival (PFS) reached 392 days + by the date of Apr. 14th, 2016.

**Conclusion:**

EHCC could be treated like orthotopic HCC by oral administration of sorafenib, which shows good tumor response and survival benefit. Delayed high fever and maculopapules are potential, rare and severe side effects of sorafenib, and could be effectively controlled by glucocorticoid.

## Background

Ectopic hepatocellular carcinoma (EHCC) is a rare malignancy from ectopic liver, a kind of developmental abnormality [1–3]. The incidence of ectopic liver is only about 0.27 – 0.7 % [3–5]. However, ectopic liver is more likely to develop primary hepatocellular carcinoma (HCC) compared with normal liver tissues. Although sorafenib is the standard first-line therapy for HCC, there is rare report on the role of sorafenib in treating EHCC [2]. Here we report a case of EHCC in the thoracic and abdominal cavities, who interestingly showed symptoms of delayed high fever and maculopapules during sorafenib treatment.

## Case presentation

**Fig. 1:**
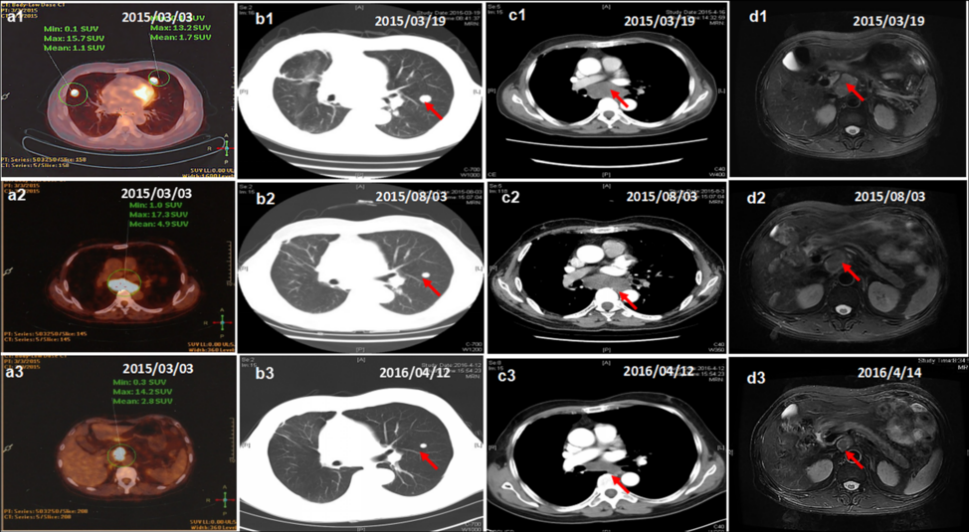
Tumor response during sorafenib treatment. PET/CT showed multiple lesions in both lungs (**a1**), mediastinal lymph nodes (**a2**) and abdominal cavity (**a3**). No lesion was found in the liver by PET/CT (*a3*), abdominal MRI (**d1**-**d3**). The tumor response in both intrathoracic and intraperitoneal lesions dramatically shrank (**b1**-**b3**, **c1**-**c3** and **d1**-**d3**)

**Fig. 2:**
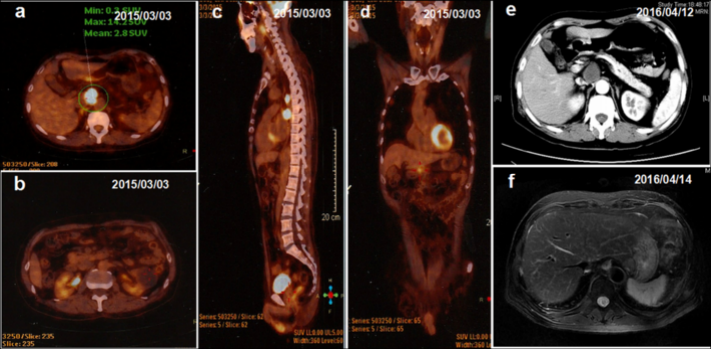
No hepatic lesion in PET-CT, abdominal CT and MRI. **a**-**d** no hepatic lesion in PET-CT at the beginning. **e**-**f** no hepatic lesion in abdominal CT and MRI at last follow-up one year later

**Fig. 3:**
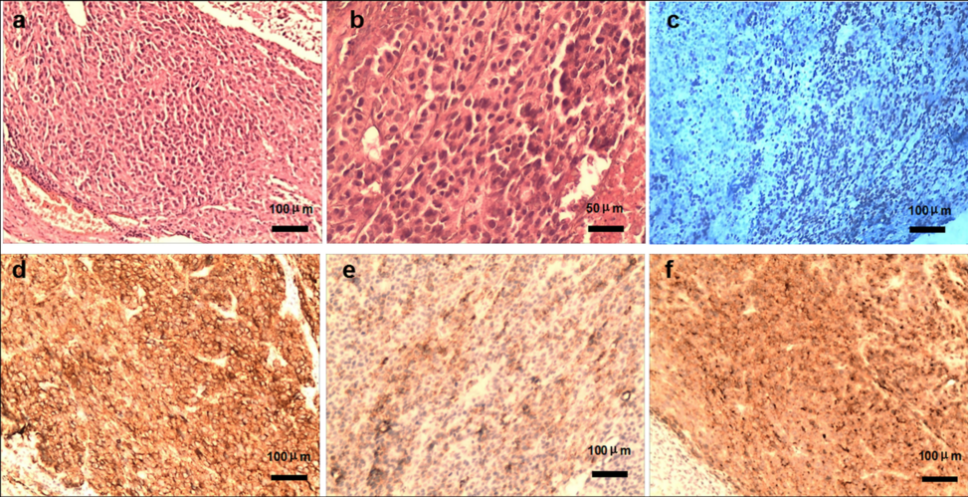
Pathological diagnosis of ectopic hepatocellular carcinoma. **a** HE staining, cancer cell nest with pseudoglandular structure and focal necrosis area (100×). **b** HE staining, morphologically typical hepatocellular carcinoma cells with polygonal shape, eosinophilic cytoplasm and big anachromasis nucleus. **c** Ki-67, positive in nucleus (60–70 %, 100×). **d** CK18, positive on membrane (++, 100×). **e** Hepatocyte, positive in cytoplasm (+, 100×). **f** Glypican-3, positive in cytoplasm (++, 100×)

A 63-year-old male presented with a history of hepatitis B virus (HBV) infection for 20 years and no anti-HBV treatment. In mid-Jan. 2015, the patient developed progressive and dull chest pain without obvious inducement. Chest CT scan revealed multiple pulmonary nodules and enlarged mediastinal lymph nodes. The level of serum alpha-fetal protein (AFP) reached 24793 ng/mL. Due to no hepatic lesion found by contrast abdominal CT and MRI scan, F-18 positron emission tomography-computed tomography (PET/CT) was carried out. Unexpectedly, PET/CT definitely showed that multiple lesions in the thoracic and abdominal cavities, including several lung nodules in the maximum size of 1.7 cm × 1.7 cm (Fig. 1 a1), mediastinal lymph nodes in the maximum size of 6.4 cm × 3.2 cm (Fig. 1 a2), and intraperitoneal mass in the fundus of stomach, cardia, portal fissure and abdominal aortas in the size of 4.6 cm × 2.2 cm (Fig. 1 a3), but still no hepatic lesion (Fig. 2a-d). On Mar. 6th, 2015, the patient underwent a wedge resection of right pulmonary. The pathological morphology displayed cancer cell nest with pseudoglandular structure and focal necrosis area, typical hepatocellular carcinoma cells with polygonal shape, eosinophilic cytoplasm and big anachromasis nucleus. And immunohistochemistry showed the expressions of CK18 ++, Glypican-3 ++, Hepatocyte +, P53 + and Ki–67 60 - 70 %, which confirmed the diagnosis of primary HCC (Fig. 3 a-f). Thus, the patient was a rare case of EHCC in an advanced stage.

**Fig. 4:**
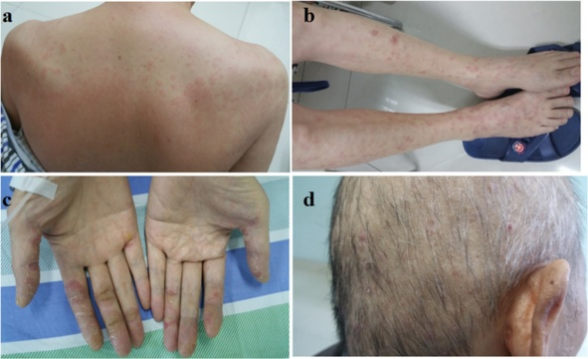
Skin reaction all over the body. Red maculopapules in the back and legs (**a**, **b**). Hand-foot skin reaction in both hands (**c**). Rash in the scalp and ears (**d**)

**Fig. 5:**
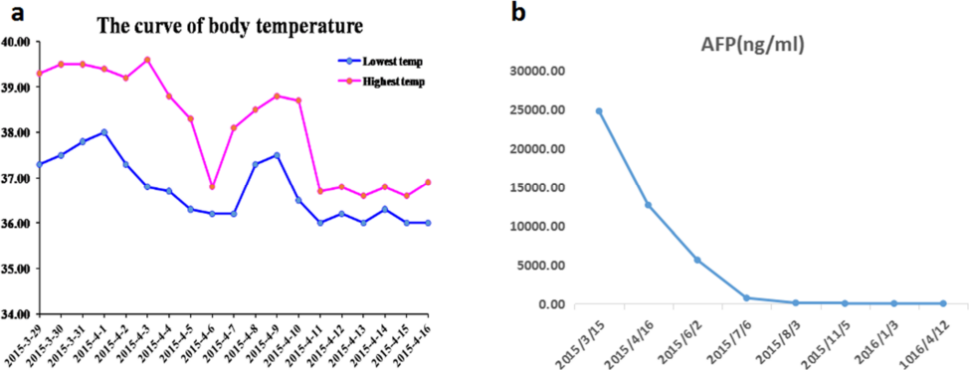
Changes in body temperature and AFP. **a** After ten-day administration of sorafenib, the patient suffered from high fever from Mar. 29th, 2015. Upon cease of sorafenib use on Apr. 3rd, 2015, the high fever decreased quickly. After taking sorafenib again at Apr. 7th, and the fever developed. After daily administration of prednisone, the body temperature returned to the normal. **b** The serum AFP gradually dropped from 24793 ng/mL to 2.19 ng/mL from March 16th, 2015 to Apr. 12th, 2016

Following the standard therapy of HCC in National Comprehensive Cancer Network (NCCN) guidelines [6], the patient began to take sorafenib 400 mg twice daily together with Chinese medicines (Jinlong capsule, Jiansheng Company, China) and immunopotentiative agents (ubenimex capsules, Yuandong Company, China) on Mar. 19th, 2015. On the tenth day (Mar. 28th, 2015), red maculopapules appeared in the face, neck, abdomen, back and legs (Fig. 4 a and b). From the same day, the patient began to suffer from continuous high fever, with highest body temperature ranging from 39.0 °C to 39.6 °C each day. The patient did not decrease the dose or stop the use of sorafenib despite feeling dizzy and fatigue. There was no abnormality in blood routine or blood culture examination, no pulmonary infection or other inflammatory signs. After taking non-steroidal drugs with antipyretic and anti-allergic properties and withdrawing all drugs except sorafenib, no significant improvement was observed in high fever and maculopapules. Therefore, the patient was asked to cease the use of sorafenib from Apr. 3rd, 2015. Strikingly, the high fever and maculopapules were alleviated quickly. On the third day, the body temperature returned to the normal level, and on the fourth day, the maculopapules almost completely disappeared. Next, the patient began to take sorafenib again at the standard dose of 400 mg twice daily on Apr. 7th, 2015. As expected, the fever developed, raising the body temperature to 38.1 °C on exactly the same day, to 38.4 °C the next day and 38.8 °C the third day. Also, the red maculopapules relapsed at the same time. Hence, prednisone was administered at a dosage of 30 mg/day on the fourth day. The body temperature was decreased and maculopapules relieved quickly. The dosage of prednisone was gradually reduced from 30 to 10 mg/day in one month, and then kept at 5 mg/day for another two weeks. After the patient’s temperature has returned to normal for more than one month, he intermittently took ubenimex and Jinlong capsule again. There has been no recurrence of fever and maculopapule to date. The dynamic change in body temperature is reflected in Fig. 5a. Other common side effects also need to be mentioned, such as hand-foot syndrome and rash in the scalp and ears and body (Fig. 4 c and d). During the follow-up, the tumor response of both intrathoracic and intraperitoneal lesions reached partial response (PR) according to RECIST 1.1 criteria. The lung nodules, mediastinal lymph nodes and intraperitoneal mass shrank gradually from Mar. 19, 2015 to Apr. 12th, 2016 (Fig. 1 b1-b3, c1-c3 and d1-d3), with the maximum size of 0.8 cm × 0.6 cm, 3.7 cm × 2.2 cm and 2.0 cm × 1.9 cm at the date of Apr. 12th, 2016, respectively. There was still no lesion in liver, revealed by repeated examinations of abdominal ultrasound, CT and MRI (Fig. 2 e and f). In addition, serum AFP gradually dropped from 24793 ng/mL to 2.19 ng/mL on Apr. 12th, 2016 following the disease control (Fig. 5b). Until Apr. 14th, 2016 in the last review, the patient had maintained a good condition with progression free survival (PFS) of 392 days + .

## Conclusions

Consistent with most HCC in orthotopic liver tissue, EHCC also arises as a result of chronic hepatitis B or C infection and secondary cirrhosis [7, 8]. However, many factors including liver metabolism disorders, damage of cellular repair, and carcinogen microenvironment, cause a greatly higher incidence of EHCC than that of orthotopic HCC [1, 4, 7]. The diagnosis of EHCC mainly depends on surgery and pathologic diagnosis, and cannot be based on serum AFP level and medical history of hepatitis B virus infection. We report a classical case of EHCC here. First, this patient suffered from a 20-year HBV infection and mild hepatocirrhosis. Second, imaging techniques showed no tumor in the liver at initial diagnosis and during the thirteen-month follow-up. Third, postoperative serum AFP level was more than 16000 ng/ml and dropped dramatically following the disease control after sorafenib treatment. Last and most important, pulmonary lobectomy provided the pathologic diagnosis of HCC.

Sorafenib is a multi-kinase inhibitor that blocks signaling pathways in tumor growth [9, 10]. In a global clinical trial of patients with advanced HCC in 2008, the median overall survival (mOS) in sorafenib group was 2.8 months longer than that in placebo group (10.7 months vs 7.9 months), and the mPFS in sorafenib group was 2.3 months longer than that in placebo group (5.5 months vs 2.8 months) [11]. And a phase III, randomised, double-blind, placebo-controlled trial in Asia-Pacific region in 2009 revealed that mOS was 2.3 months longer in sorafenib group than in placebo group (6.5 months vs 4.2 months) [12]. In the current case, the patient keeps a good condition with PFS of 392 days+, which is much longer than that in previous trials [11, 12]. To the best of our knowledge, this is the first case report that describes sorafenib treatment in a case of EHCC.

Sorafenib also produces adverse effects including skin toxicity, gastrointestinal reaction, systemic reaction and vascular dysfunction, hoarseness, fever, pain, ulcers in the mouth, etc. Hand-foot syndrome and rash are the most common adverse events. In the Sorafenib Hepatocellular Carcinoma Assessment Randomized Protocol (SHARP) trial, hand-foot skin reaction occurred in up to 21 % of enrolled patients [13]. Sorafenib treatment in HCC possibly causes rare side effects, such as rhabdomyolysis thyroid crisis and liver failure [14–17]. Most side effects could be alleviated after reducing or ceasing the use of the drug. To the best of our knowledge, this is the first case report that describes delayed high fever and maculopapules during sorafenib treatment as well. High fever and maculopapules rapidly relieved when ceasing sorafenib treatment and returned soon after sorafenib re-challenging. We propose that the delayed high fever and maculopapules are potential side effects of sorafenib. Additionally, we can exclude the cause of delayed high fever in the use of ubenimex and Jinlong capsule, because we stopped these medicines once the patient suffered from fever. However, there was no change in high fever. And when the patient took these medicine later, there has been no recurrence of fever.

In conclusion, EHCC could be treated like orthotopic HCC by orally administering sorafenib, and the treatment can achieve good tumor response and survival benefit. Delayed high fever and maculopapules are potential, rare and severe side effects of sorafenib, which could be effectively controlled by glucocorticoid.

## Abbreviations

AFP, alpha-fetal protein; EHCC, ectopic hepatocellular carcinoma; HBV, hepatitis B virus; HCC, hepatocellular carcinoma; NCCN, National Comprehensive Cancer Network; PET/CT, positron emission tomography-computed tomography; PFS, progression free survival

## References

[1] Yamashita M, Nagamine Y, Ozaki K, Ueshima S, Takahashi H, Inoue H (1985). An autopsy case of a cirrhotic ectopic liver with a review of the literature. Acta Hepatol Jpn.

[2] Pavel Z, Lubomir M, Peter I, Jan F (2013). Ectopic liver: different manifestations, one solution. World J Gastroenterol.

[3] Arakawa M, Kimura Y, Sakata K, Kubo Y, Fukushima T, Okuda K (1999). Propensity of ectopic liver to hepatocarcinogenesis: case reports and a review of the literature. Hepatology.

[4] Watanabe M, Matsura T, Takatori Y, Ueki K, Kobatake T, Hidaka M (1989). Five cases of ectopic liver and a case of accessory lobe of the liver. Endoscopy.

[5] Sato S, Watanabe M, Nagasawa S, Niigaki M, Sakai S, Akagi S (1998). Laparoscopic observations of congenital anomalies of the liver. Gastrointest Endosc.

[6] Cainap C, Qin S, Huang WT, Chung IJ, Pan H, Cheng Y (2015). Linifanib versus Sorafenib in patients with advanced hepatocellular carcinoma: results of a randomized phase III trial. J Clin Oncol.

[7] Caygill CP, Gatenby PA (2004). Ectopic liver and hepatocarcinogenesis. Eur J Gastroenterol Hepatol.

[8] Asselah T, Condat B, Cazals-Hatem D, Hassani Z, Bernuau J, Groussard O (2001). Ectopic hepatocellular carcinoma arising in the left chest wall: a long-term follow-up. Eur J Gastroenterol Hepatol.

[9] Wilhelm SM, Carter C, Tang L, Wilkie D, McNabola A, Rong H (2004). BAY 43–9006 exhibits broad spectrum oral antitumor activity and targets the RAF/MEK/ERK pathway and receptor tyrosine kinases involved in tumor progression and angiogenesis. Cancer Res.

[10] Carlomagno F, Anaganti S, Guida T, Salvatore G, Troncone G, Wilhelm SM (2006). BAY 43–9006 inhibition of oncogenic RET mutants. J Natl Cancer Inst.

[11] Llovet JM, Ricci S, Mazzaferro V, Hilgard P, Gane E, Blanc JF (2008). SHARP Investigators Study Group. Sorafenib in advanced hepatocellular carcinoma. N Engl J Med.

[12] Cheng AL, Kang YK, Chen Z, Tsao CJ, Qin S, Kim JS (2009). Efficacy and safety of sorafenib in patients in the Asia-Pacific region with advanced hepatocellular carcinoma: a phase III randomised, double-blind, placebo-controlled trial. Lancet Oncol.

[13] Rimassa L, Santoro A (2009). Sorafenib therapy in advanced hepatocellular carcinoma: the SHARP trial. Expert Rev Anticancer Ther.

[14] Tsuji K, Takemura K, Minami K, Teramoto R, Nakashima K, Yamada S (2013). A case of rhabdomyolysis related to sorafenib treatment for advanced hepatocellular carcinom. Clin J Gastrornterol.

[15] Haraldsdottir S, Li Q, Villalona-Calero MA, Olencki TE, Kendra K, Ing SW (2013). Case of sorafenib-induced thyroid storm. J Clin Oncol.

[16] Hootegem AV, Verslype C, Van Steenbergen WV (2011). Sorafenib-induced liver failure: a case report and review of the literature. Case Reports Hepatol.

[17] Takeda H, Nishikawa H, Iguchi E, Matsuda F, Kita R, Kimura T (2012). Sorafenib-induced acute interstitial pneumonia in patients with advanced hepatocellular carcinoma: report of three cases. Clin J Gastroenterol.

